# Anti-tubercular Drugs Dominate Drug-Induced Liver Injury in Northern India: An Observational, Cross-Sectional Study of 100 Cases Using the Revised Electronic Causality Assessment Method (RECAM) and Drug-Induced Liver Injury Network (DILIN) Criteria

**DOI:** 10.7759/cureus.105041

**Published:** 2026-03-11

**Authors:** Fatima Rani, Ranjan Awana, Ajay Patwa, Suyog Sindhu, Ajay Verma, Sarvesh Singh

**Affiliations:** 1 Pharmacology and Therapeutics, King George's Medical University, Lucknow, IND; 2 Gastroenterology and Hepatology, King George’s Medical University, Lucknow, IND; 3 Pharmacology, King George's Medical University, Lucknow, IND; 4 Respiratory Medicine, King George's Medical University, Lucknow, IND; 5 Pulmonary Critical Care Medicine, King George's Medical University, Lucknow, IND; 6 Experimental Pharmacology, King George’s Medical University, Lucknow, IND

**Keywords:** anti-tubercular drugs, comorbidities, dilin, drug-induced liver injury, recam

## Abstract

Background: Drug-induced liver injury (DILI) is a major cause of acute liver failure in India, yet prospective data from northern India are rare. We analyzed drugs, clinical patterns, severity, and risk factors associated with DILI in a tertiary care hospital.

Methods: This observational, cross-sectional study (IEC letter no. XXII-PGTSC-IIA/P9; March 2023-February 2024) included 100 patients with DILI (alanine transaminase (ALT)≥5×upper limit of normal (ULN) or ALT≥3×ULN plus bilirubin≥2×ULN). Causality assessment was performed using the Revised Electronic Causality Assessment Method (RECAM), and severity was classified by the Drug-Induced Liver Injury Network (DILIN) scale. Multivariate logistic regression identified predictors of moderate-to-severe DILI (grades 3-5).

Results: Average age was 52.6±16.1 years, with 51% male. Anti-tubercular therapy (ATT) was implicated in 39 (39%) cases, antibiotics in 20 (20%), antiepileptics in 17 (17%), and complementary/alternative medicines (CAM) in 7 (7%) cases. Hepatocellular injury was the most common pattern, observed in 46 (46%) patients, followed by mixed injury in 36 (36%) and cholestatic injury in 18 (18%) patients. Regarding severity, 58 (58%) patients had Grade 1+, 19 (19%) had Grade 2+, 15 (15%) had Grade 3+, 6 (6%) had Grade 4+, and 2 (2%) had Grade 5+ injury. Hypertension (adjusted odds ratio (aOR) 4.14; 95% CI 1.65-57.40), diabetes (aOR 2.18; 95% CI 1.30-10.80), and CAM use (aOR 1.80; 95% CI 1.20-7.32) were independently associated with moderate-to-severe DILI. No clinical variable significantly predicted injury pattern.

Conclusions: ATT remains the foremost DILI trigger in northern India. Metabolic comorbidities and CAM use were associated with increased severity. RECAM-guided causality and DILIN grading enhance bedside risk stratification.

## Introduction

Drug-induced liver injury (DILI) is an important public health concern worldwide, accounting for up to 10% of all cases of acute liver failure (ALF) [1] and representing the most frequent cause of post-marketing drug withdrawal [2]. The liver, being the primary site of xenobiotic metabolism, is susceptible to both predictable (intrinsic) and unpredictable (idiosyncratic) hepatotoxicity [3]. Intrinsic injury, for example by acetaminophen overdose, occurs in a dose-dependent fashion via toxic metabolites such as N-acetyl-p-benzoquinone imine (NAPQI) [4], whereas idiosyncratic injury is dose-independent and linked to host factors like genetic polymorphisms in metabolizing enzymes such as N-acetyltransferase 2 (NAT2) and cytochrome P450 2E1 (CYP2E1) [5,6], human leukocyte antigen (HLA) alleles [7], and environmental cofactors such as alcohol or malnutrition [8].

Globally, population-based studies report annual DILI incidence ranging from one to 19 per 100,000 inhabitants [9]. In Western registries, antimicrobials and herbal supplements are leading culprits [10]; however, data from India reveal a distinct profile dominated by anti-tubercular therapy (ATT), antiepileptics, and unregulated complementary and alternative medicine (CAM) [11]. A recent multicentric Indian survey identified ATT in 39-49% of confirmed DILI cases, with up to 25% developing moderate-to-severe hepatotoxicity [11]. Despite this burden, prospective, systematically adjudicated data from northern India remain scarce, hampering region-specific guidelines.

Clinical presentation spans asymptomatic transaminitis to liver failure, including both ALF and acute-on-chronic liver failure (ACLF) [12,13]. Biochemical injury patterns, hepatocellular, cholestatic, or mixed, are classified by the R-value and influence prognosis [14]. Severity is graded by the Drug-Induced Liver Injury Network (DILIN) scale, and Hy’s law (alanine transaminase (ALT)>3×upper limit of normal (ULN)+bilirubin>2×ULN) predicts a 10-50% risk of death or transplantation [1,15]. Yet, few studies integrate validated causality tools such as the Revised Electronic Causality Assessment Method (RECAM) [16,17] with severity grading in Indian populations.

Metabolic comorbidities such as diabetes mellitus, hypertension, and obesity are hypothesized to exacerbate DILI by amplifying oxidative stress and impairing hepatic regeneration [18]. Concurrent use of CAM, often undisclosed by patients, further complicates causality attribution and increases hepatotoxic risk [19]. In this context, we conducted an observational cross-sectional study to better characterize the burden and determinants of DILI in our setting. The objectives of this study were to (1) identify the most commonly implicated drugs causing DILI in a tertiary care setting in northern India, (2) characterize the clinical and biochemical patterns of injury, (3) assess severity using the DILIN grading system, and (4) identify clinical risk factors associated with moderate-to-severe DILI.

## Materials and methods

Study design and setting

This single-center, cross-sectional, observational study was conducted from March 2023 to February 2024 in the departments of Pharmacology & Therapeutics, Medicine, and Respiratory Medicine at King George’s Medical University, Lucknow, Uttar Pradesh, India. Institutional Ethics Committee (IEC letter no. XXII-PGTSC-IIA/P9) approval was obtained, and written informed consent was obtained from each participant.

Participants

Consecutive patients aged 18 years or older presenting to the medicine or respiratory medicine departments (outpatient or inpatient) with suspected acute liver injury were screened for eligibility. Diagnosis required meeting at least one of the following biochemical criteria [18]: ALT≥5×ULN, or alkaline phosphatase (ALP)≥2×ULN, or ALT≥3×ULN with total bilirubin≥2×ULN.

Exclusion criteria were pregnancy, lactation, viral hepatitis (hepatitis A virus (HAV), hepatitis B virus (HBV), hepatitis C virus (HCV), and hepatitis E virus (HEV)), autoimmune hepatitis, Wilson disease, alcohol excess (>40 g/day for men or >20 g/day for women), sepsis, malignancy, biliary obstruction, or inadequate drug-exposure history.

Data collection

A standardized case-record form captured demographics (age, sex, and body mass index), comorbidities (diabetes, hypertension, and hypothyroidism), detailed prescription history, over-the-counter drugs, and CAM. Latency (days from first dose to symptom onset), clinical features (jaundice, ascites, and hepatic encephalopathy), laboratory tests (ALT, aspartate aminotransferase (AST), ALP, total/direct bilirubin, international normalized ratio (INR), albumin, creatinine, and electrolytes), and imaging (ultrasound/CT) were documented. Baseline pre-exposure liver function tests were not uniformly available for all patients and, therefore, were not included in the comparative analysis. Cases with incomplete essential clinical information, drug-exposure history, or laboratory parameters required for causality and severity assessment were excluded during the initial screening process. Analyses were conducted using complete-case data without imputation.

RECAM scoring was applied using the predefined structured domains and standardized criteria described in the instrument to ensure uniform causality categorization across cases. It was applied to all suspected DILI cases, and its structured, algorithmic format helped standardize assessment of latency, de-challenge, risk factors, and competing etiologies. Causality was classified as definite, highly likely, probable, possible, or unlikely. Injury patterns were identified by the R-ratio [20]: R>5: hepatocellular, 2<R≤5: mixed, R≤2: cholestatic.

Severity was graded using the DILIN scale [21]. Grade 1+ (mild) was defined as asymptomatic liver injury without evidence of hepatic dysfunction. Grade 2+ (moderate) included symptomatic cases without features of liver failure. Grade 3+ (moderate-severe) comprised patients requiring hospitalization and/or demonstrating biochemical evidence of coagulopathy (elevated INR) or clinical features such as hepatic encephalopathy. Grade 4+ (severe) corresponded to cases fulfilling criteria for ALF, and Grade 5+ indicated liver transplantation or death. Severity grading was assigned based on clinical evaluation and laboratory parameters at presentation.

Statistical analysis

Data were analyzed using SPSS v21 (IBM, Armonk, NY). Descriptive statistics were expressed as means±SD or frequencies (%). Risk factors for moderate-to-severe DILI (grades 3-5) were evaluated by binary logistic regression; predictors of injury pattern were explored via multinomial logistic regression with hepatocellular injury as the reference. Variables entered into the multivariate logistic regression model included diabetes mellitus, hypertension, and use of CAM. These were selected based on clinical plausibility and findings from univariate analysis. A p-value <0.05 was considered significant.

## Results

Out of 112 screened patients, 100 met the inclusion criteria. The 12 excluded patients had alternative diagnoses or incomplete histories. The mean age of the included cohort was 52.6±16.1 years, with 51% being male. The mean latency period was 48.5±32.9 days. All patients received standard recommended therapeutic doses according to prevailing treatment guidelines. Drug-exposure duration beyond latency was not independently analyzed as a determinant of case clustering. Comorbid conditions were identified in 68% of patients, most commonly diabetes mellitus (29%), hypertension (31%), and hypothyroidism (8%). Furthermore, 23% of patients reported concomitant use of CAM products (Table 1).

**Table 1:** Demographic, clinical, and laboratory characteristics of 100 DILI patients BMI, body mass index; CAM, complementary and alternative medicine; ALT, alanine aminotransferase; AST, aspartate aminotransferase; ALP, alkaline phosphatase; INR, international normalized ratio; MELD, model for end-stage liver disease

Characteristic	Mean±SD or n (%)
Age (years)	52.6±16.1
Male sex	51 (51%)
BMI (kg/m²)	24.6±4.1
Comorbidities	
Diabetes mellitus	29 (29%)
Hypertension	31 (31%)
Hypothyroidism	8 (8%)
Concomitant CAM use	23 (23%)
Latency (days)	48.5±32.9
Laboratory parameters at presentation	
ALT (IU/L)	619.2±313
AST (IU/L)	509.2±283
ALP (IU/L)	355.2±145
Total bilirubin (mg/dL)	11.8±7.0
INR	2.6±0.9
MELD score	17.4±5.6

ATT (isoniazid, rifampicin, pyrazinamide, and ethambutol (HRZE)) was the most common class in 39 (39%) cases, followed by antibiotics in 20 (20%), antiepileptics in 17 (17%), and CAM formulations in 7 (7%) patients. Among individual agents, isoniazid-rifampicin-pyrazinamide-ethambutol combinations predominated in 31 (31%), followed by phenytoin in 9 (9%) and amoxicillin-clavulanate in 7 (7%) cases (Table 2).

**Table 2:** Frequency of implicated drug classes in DILI cases (n=100) ATT, anti-tubercular therapy; CAM, complementary and alternative medicine

Implicated drug class	Frequency (n)	Percentage (%)
ATT	39	39%
Antibiotics	20	20%
Antiepileptics	17	17%
Miscellaneous	15	15%
CAM (herbal, unani, homeopathic, etc.)	7	7%
Antifungals	2	2.0%

**Figure 1:**
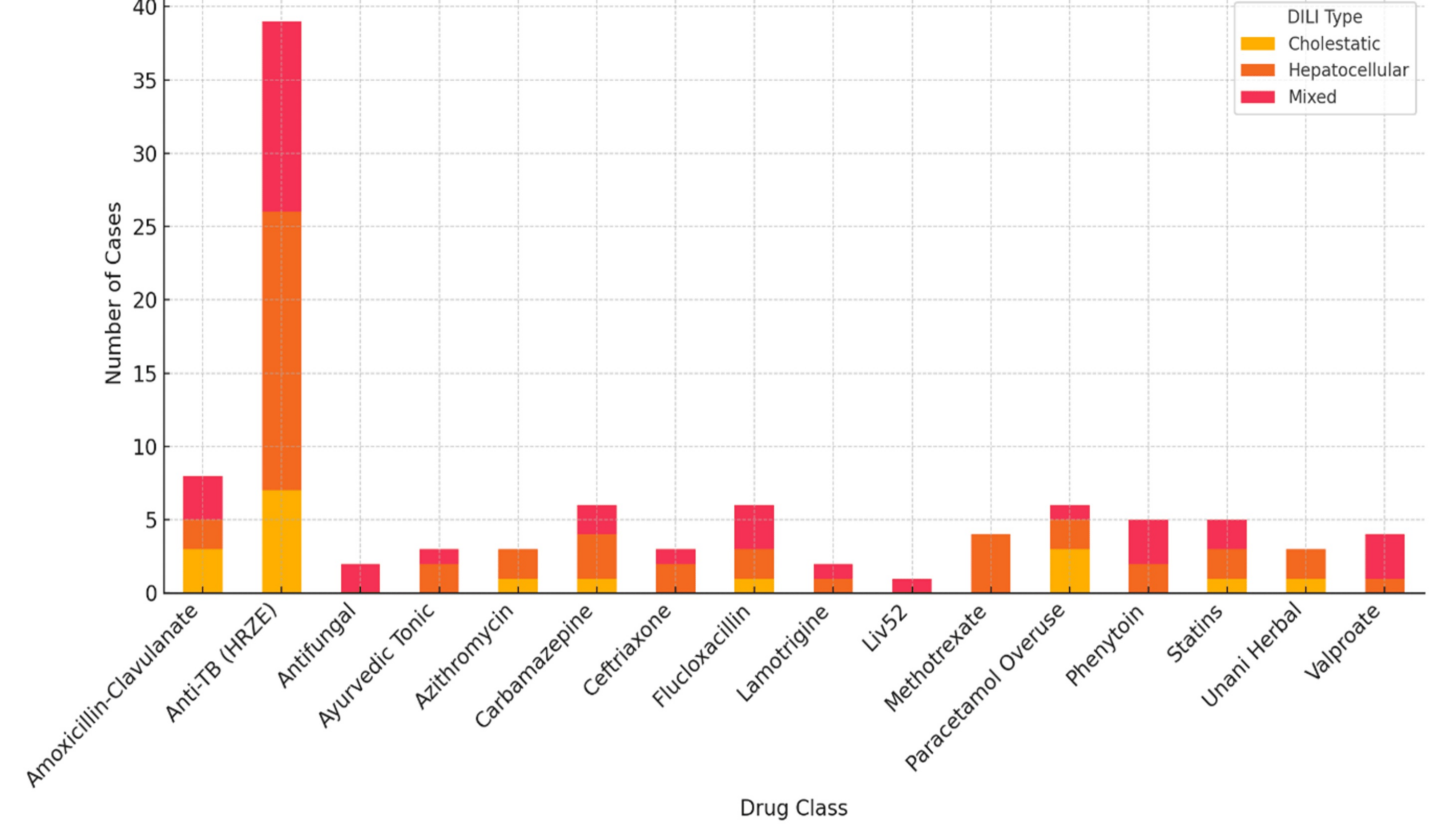
Distribution of DILI type across drug classes HRZE, isoniazid, rifampicin, pyrazinamide, and ethambutol; DILI, drug-induced liver injury

Hepatocellular injury was observed in 46 (46%) cases, mixed in 36 (36%), and cholestatic in 18 (18%). ATT exhibited both hepatocellular and mixed patterns (48.7% vs. 33.3%), whereas amoxicillin-clavulanate showed balanced cholestatic and mixed presentations (37.5% each). Methotrexate exclusively produced hepatocellular injury (100%, Figure 1).

**Figure 2:**
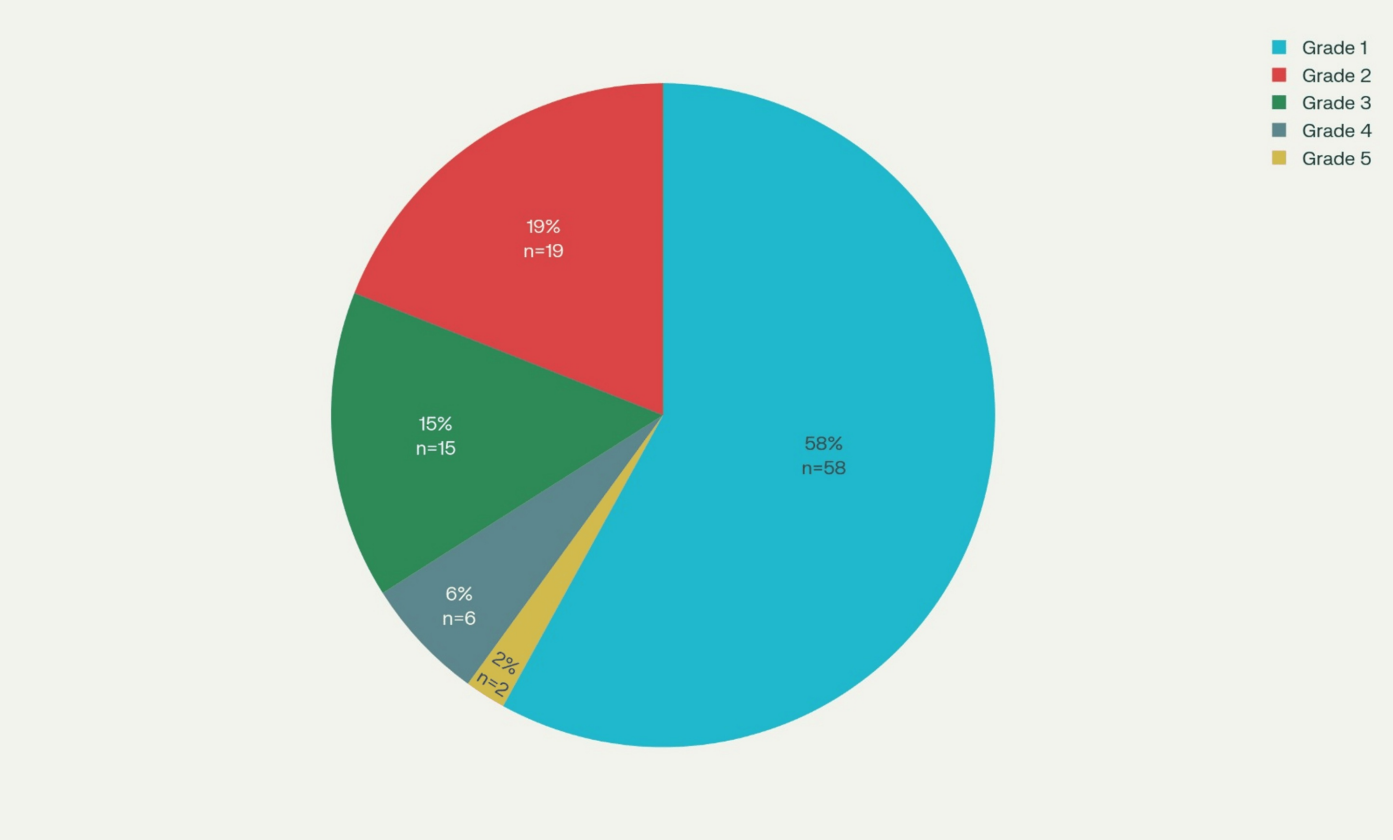
Pie chart showing the overall distribution of DILI severity grades DILI, drug-induced liver injury

Grade 1+ (mild) occurred in 58 (58%) patients, Grade 2+ (moderate) in 19 (19%), Grade 3+ (moderate-severe) in 15 (15%), Grade 4+ (severe) in 6 (6%), and Grade 5+ (fatal/transplant) in 2 (2%) patients (Figure 2). ATT accounted for the largest number of Grade 3+ cases (17.9%), including one fatal outcome. Among antiepileptic agents, phenytoin and valproate demonstrated higher proportions of severe injury, with Grade 3+ injury in 40% of phenytoin-exposed patients and Grade 3+ and Grade 4+ injury in 75% of valproate-exposed patients. Carbamazepine was associated with one fatal case (Grade 5+). Other agents, including paracetamol overuse, azithromycin, and lamotrigine, contributed to severe presentations in smaller numbers (Table 3).

**Table 3:** Distribution of DILI severity grades across implicated drug classes ATT, anti-tubercular therapy; HRZE, isoniazid, rifampicin, pyrazinamide, ethambutol

Drug class/agent	Grade 1+ (n, %)	Grade 2+ (n, %)	Grade 3+ (n, %)	Grade 4+ (n, %)	Grade 5+ (n, %)
ATT (HRZE)	25 (64.1%)	5 (12.8%)	7 (17.9%)	1 (2.6%)	1 (2.6%)
Amoxicillin and clavulanate	4 (50.0%)	3 (37.5%)	0 (0.0%)	1 (12.5%)	0 (0.0%)
Phenytoin	3 (60.0%)	0 (0.0%)	2 (40.0%)	0 (0.0%)	0 (0.0%)
Carbamazepine	2 (33.3%)	3 (50.0%)	0 (0.0%)	0 (0.0%)	1 (16.7%)
Methotrexate	1 (25.0%)	2 (50.0%)	1 (25.0%)	0 (0.0%)	0 (0.0%)
Statins	4 (80.0%)	1 (20.0%)	0 (0.0%)	0 (0.0%)	0 (0.0%)
Paracetamol overuse	4 (66.7%)	0 (0.0%)	1 (16.7%)	1 (16.7%)	0 (0.0%)
Valproate	1 (25.0%)	0 (0.0%)	2 (50.0%)	1 (25.0%)	0 (0.0%)
Azithromycin	0 (0.0%)	1 (33.3%)	1 (33.3%)	1 (33.3%)	0 (0.0%)
Flucloxacillin	3 (50.0%)	3 (50.0%)	0 (0.0%)	0 (0.0%)	0 (0.0%)
Ceftriaxone	3 (100.0%)	0 (0.0%)	0 (0.0%)	0 (0.0%)	0 (0.0%)
Ayurvedic tonic	3 (75%)	1 (25%)	0 (0.0%)	0 (0.0%)	0 (0.0%)
Unani herbal	3 (100.0%)	0 (0.0%)	0 (0.0%)	0 (0.0%)	0 (0.0%)
Antifungal	1 (50.0%)	0 (0.0%)	1 (50.0%)	0 (0.0%)	0 (0.0%)
Lamotrigine	1 (50.0%)	0 (0.0%)	0 (0.0%)	1 (50.0%)	0 (0.0%)
Total	58 (58%)	19 (19%)	15 (15%)	6 (6%)	2 (2%)

Binary logistic regression identified hypertension (adjusted odds ratio (aOR) 4.14; 95% CI 1.65-57.40), diabetes (aOR 2.18; 95% CI 1.30-10.80), and CAM use (aOR 1.80; 95% CI 1.20-7.32) as independent predictors (Table 4). No variable significantly predicted injury pattern in multinomial analysis.

**Table 4:** Independent predictors of moderate-to-severe DILI (binary logistic regression) DILI, drug-induced liver injury; CAM, complementary and alternative medicine

Risk factor	Adjusted OR	95% CI	p-value
Hypertension	4.14	1.65-57.40	0.008
Diabetes mellitus	2.18	1.30-10.80	0.014
CAM use	1.80	1.20-7.32	0.021

Overall, 87 (87%) patients recovered, 7 (7%) had ongoing injury, 4 (4%) underwent liver transplantation, and 2 (2%) died. Severe outcomes included ALF in 8 (8%) patients, ICU admission in 13 (13%), and mechanical ventilation in 7 (7%). Liver transplantation was performed in 4 (4%) patients, and overall mortality was observed in 2 (2%).

## Discussion

Our evaluation of 100 DILI patients in a North Indian tertiary center confirms ATT as the dominant culprit in 39 (39%) patients, followed by antibiotics and antiepileptics. As ATT was administered as standard multidrug regimens, this proportion reflects regimen-level exposure rather than a direct comparison with single-drug categories. The predominance of ATT mirrors findings from national pharmacovigilance reports [11,22] and reflects the high burden of tuberculosis in India. Notably, antiepileptic agents in 17 (17%) cases and the use of CAM in 7 (7%) also contribute substantially, highlighting the need for more rigorous regulatory supervision and patient education.

Clinical heterogeneity included 46 (46%) cases of hepatocellular, 36 (36%) mixed, and 18 (18%) cholestatic patterns. ATT showed a hepatocellular/mixed spectrum that corresponds to metabolite-mediated toxicity (e.g., hydrazine from isoniazid) [23] and immune-mediated mechanisms [24], which are known to produce histopathological lesions such as ballooning degeneration and necrosis, as demonstrated in studies of toxic liver injury [25]. In contrast, amoxicillin-clavulanate displayed a cholestatic/mixed profile, which is consistent with its known inhibition of bile-acid transporters [26]. These phenotype-drug correlations validate RECAM-guided classification and emphasize pattern-specific monitoring strategies.

Severity assessment using the DILIN grading scale revealed that 23% of patients progressed to moderate-to-severe injury (grades 3-5). ATT accounted for the largest number of severe cases in absolute terms, while certain antiepileptic agents demonstrated higher proportions of severe injury within their respective exposure groups. This mirrors DILIN data, where phenytoin and valproate have been frequently associated with ALF [27]. However, these subgroup observations are based on small numbers and should be interpreted cautiously. Fatal outcomes were observed within the ATT and antiepileptic cohorts, underscoring the clinical seriousness of DILI in these settings.

Multivariate logistic regression demonstrated that hypertension (aOR 4.14; 95% CI 1.65-57.40), diabetes (aOR 2.18; 95% CI 1.30-10.80), and CAM use (aOR 1.80; 95% CI 1.20-7.32) were associated with increased odds of moderate-to-severe DILI. The wide confidence intervals, particularly for hypertension, reflect limited precision attributable to the modest number of severe events and warrant cautious interpretation. Metabolic comorbidities enhance oxidative stress, impair mitochondrial resilience, and modulate drug metabolism, thereby amplifying hepatocyte injury [28,29]. CAM use, despite presenting mostly as mild injury (grade 1+), was associated with 1.8-fold higher odds of severe outcomes, most likely due to underreporting, polypharmacy interactions, and product adulteration [30].

This study has several limitations. The single-center design and modest sample size limit generalizability. As a tertiary referral center, referral bias may have resulted in overrepresentation of more severe cases. Baseline pre-exposure liver function tests were not uniformly available, and cumulative dose or duration of exposure was not analyzed as an independent predictor. The relatively small number of severe cases contributed to wide confidence intervals in regression modeling. Recall bias regarding CAM exposure is also possible. Furthermore, the absence of liver biopsy or genetic susceptibility testing limits mechanistic confirmation.

Nevertheless, the structured application of RECAM, as opposed to unstructured clinical judgment alone, enhanced diagnostic consistency and enabled uniform categorization of cases into definite, highly likely, probable, or possible DILI. Compared with the traditional Roussel Uclaf Causality Assessment Method (RUCAM), RECAM provides a digitized and standardized framework with clearer definitions for competing etiologies and concomitant drug exposure, thereby minimizing computational errors and subjective variability. In a setting characterized by polypharmacy and frequent complementary medicine use, this structured approach improves reproducibility and transparency in causality adjudication while preserving the central role of expert clinical evaluation. Together with DILIN severity grading and systematic comorbidity profiling, this methodology strengthens diagnostic rigor and supports applicability to similar resource-constrained environments.

Overall, this study confirms ATT as the principal cause of DILI in this North Indian cohort and identifies underlying metabolic comorbidities (hypertension and diabetes) and concurrent CAM use as significant risk factors for progression to more severe liver injury. Regular evaluation with RECAM-based causality assessment and severity grading in routine clinical practice, coupled with increased vigilance in patients with diabetes and high blood pressure, can reduce adverse liver outcomes and inform field-specific pharmacovigilance strategies.

## Conclusions

ATT is the leading cause of DILI in northern India, often presenting as hepatocellular or mixed injury. Diabetes, hypertension, and unsupervised CAM use were independently associated with an increased risk of moderate-to-severe hepatotoxicity. The application of RECAM and DILIN criteria routinely at the bedside can guide early de-challenge, targeted monitoring, and policy-driven regulation of high-risk therapies.
